# Tracking the Emergence of the Consonant Bias in Visual-Word Recognition: Evidence with Developing Readers

**DOI:** 10.1371/journal.pone.0088580

**Published:** 2014-02-11

**Authors:** Ana Paula Soares, Manuel Perea, Montserrat Comesaña

**Affiliations:** 1 Human Cognition Lab, CIPsi, School of Psychology, Universidade do Minho, Braga, Portugal; 2 ERI-Lectura and Departamento de Metodología, Universitat de València, Valencia, Spain; 3 Basque Center on Brain, Language, and Cognition, Donostia-San Sebastián, Spain; 4 Cognitive Processes and Behaviour Research Group, University of Santiago de Compostela, Santiago de Compostela, Spain; University of Akron, United States of America

## Abstract

Recent research with skilled adult readers has consistently revealed an advantage of consonants over vowels in visual-word recognition (i.e., the so-called “consonant bias”). Nevertheless, little is known about how early in development the consonant bias emerges. This work aims to address this issue by studying the relative contribution of consonants and vowels at the early stages of visual-word recognition in developing readers (2^nd^ and 4^th^ Grade children) and skilled adult readers (college students) using a masked priming lexical decision task. Target words starting either with a consonant or a vowel were preceded by a briefly presented masked prime (50 ms) that could be the same as the target (e.g., *pirata*-*PIRATA* [pirate-PIRATE]), a consonant-preserving prime (e.g., *pureto*-*PIRATA*), a vowel-preserving prime (e.g., *gicala*-*PIRATA*), or an unrelated prime (e.g., *bocelo* -*PIRATA*). Results revealed significant priming effects for the identity and consonant-preserving conditions in adult readers and 4^th^ Grade children, whereas 2^nd^ graders only showed priming for the identity condition. In adult readers, the advantage of consonants was observed both for words starting with a consonant or a vowel, while in 4^th^ graders this advantage was restricted to words with an initial consonant. Thus, the present findings suggest that a Consonant/Vowel skeleton should be included in future (developmental) models of visual-word recognition and reading.

## Introduction

The written symbols that represent the sounds of spoken language in the Greek/Latin alphabets (i.e., consonants and vowels) can be traced back to the Phoenician script (an extinct Semitic language), in which only the consonants were represented in print. Nowadays, other Semitic languages like Hebrew or Arabic only employ consonantal information in writing, except for some long vowels. This may be taken to suggest that consonants may be more important than vowels during lexical access. Indeed, as a large number of experiments conducted in different languages have consistently found, skilled adult readers rely more on consonants than on vowels during visual-word recognition and reading [1]–[12].

New at al. [8] reported an elegant and conclusive demonstration of the advantage of consonantal over vocalic information at the early stages of visual-word recognition with skilled adult readers in French. Using a lexical decision task with briefly presented (50 ms) forwardly masked primes, the authors employed nonword primes that preserved either the consonants (e.g., duvo) or the vowels (e.g., rifa) of the target words (e.g., DIVA) – identical (e.g., diva-DIVA) and unrelated (e.g., rufo-DIVA) nonword primes were also introduced as controls. They found faster recognition times for words preceded by a consonant-preserving prime than for words preceded by a vowel-preserving prime (i.e., duvo-DIVA faster than rifa-DIVA), whereas word recognition times in the vowel-preserving condition were comparable to those observed in the unrelated priming condition (see also [5] for converging evidence using subset primes: csn-CASINO faster than aio-CASINO). New et al. [8] concluded that “lexical representations are accessed more reliably through consonantal than vocalic information” (p. 1226), and established the presence of a consonant bias at early stages of visual-word recognition in adult readers.

Despite the strong evidence of an advantage of consonants over vowels at early stages of visual-word recognition in skilled adult readers, little is known about how early in development the consonant bias emerges and evolves during reading acquisition. The experiments reported in this paper address this issue being, to the best of our knowledge, the first that aims to track the emergence of the consonant bias at the early stages of visual-word recognition in developing readers. Although there is no empirical evidence about the emergence of the consonant bias in reading acquisition, a substantial amount of research has explored how infants and children process consonants and vowels in speech. Nazzi [13] using a name-based categorization paradigm (in which children are taught two different labels for three name-object pairs - two of them sharing the same label [e.g., /pize/] and the third object has a different label [e.g., /tize/] – and then are asked to put together the objects that share the same label), was one of the first authors to show that 20-month-old children are able to distinguish between two labels of the name-object pairs that differ by one consonant (e.g., /pize/- /tize/), but failed to learn the name-object pairs that differ by one vowel (e.g., /pize/- /pyze/). The advantage of consonants over vowels in word learning was found to be independent of the type of consonants used (plosives [13]; fricatives, liquids, and nasals [14]) and of their position in the speech signal (onset: /pod/–/bod/; coda: /pod/–/pot/- [13], [15]). Other studies have provided similar evidence with younger- (14-month-olds [16]; 16-month-olds [17]; 17-month-olds [18]) and older-infants (30-month-olds [19]), and preschool children (3 to 5 year-olds [20]). Moreover, measuring the comprehension of correct and mispronounced instances of familiar words (e.g., /bal/) in toddlers, several studies [21]-[24] found that the recognition of mispronounced familiar words was more impaired after a consonant transformation (e.g., /bal/–/gal/) than after a vowel transformation (e.g., /bal/–/bul/) and this result seems to be independent of the position (onset vs. coda) the consonant change occurs in [25].

Taken together, the above-cited studies show that the consonant bias in speech emerges early in development. That is, infants rely more on consonants than on vowels when they have to code lexicalized forms and/or recognize familiar stimuli. One remaining question is whether the advantage of consonants over vowels observed early in speech would also be observed at early stages of reading acquisition, i.e., when children enter school and formally start to learn how to read. Reading is a very complex cognitive process that involves the ability to construct linguistic meaning from written representations of language. When children start reading they need to learn to map the written forms onto the existing spoken word representations to access word meanings and to “know” (at least in alphabetic languages) that changes in a single letter may imply the existence of different words in the lexicon (e.g., invern vs. infern). Children are very skillful in this process and progress very quickly from an initial stage of sublexical letter-by-letter phonological recoding to a whole-word orthographic recognition process (allowed by the development of a specialized system of parallel letter processing) which is thought to constitute the main pathway to access meaning from print in skilled readers [26]–[28]. Of particular interest here is the Consonant-Vowel hypothesis developed by Nespor et al. [29], which states that consonants are more informative for lexical distinctions in lexical access, whereas vowels are more informative for (morpho)syntactic processing. This functional dissociation, which has been supported by several studies - see, for example [30]–[33], was initially proposed to explain the difficulties that young children encounter when they have to simultaneously learn the lexical and syntactic properties of words. The rationale here is that dividing the labor of consonants/vowels at two linguistic levels would be less demanding for children and more effective in child language acquisition than using both consonants and vowels to learn the lexical and syntactic information at the same time [29]. Therefore, the presence of a consonant bias at early stages of reading acquisition in our study would provide additional support to the functional distinction between consonants and vowels in language processing, thus extending the results previously observed in speech with infants and children to young readers.

Furthermore, developmental evidence on the emergence of the consonant bias in reading acquisition may also help build and constraint future implementations of computational models of visual-word recognition. Although it is generally recognized that, at some point in processing, the recognition of a printed word is mediated by the consonant-vowel status of its letters (i.e., the graphemic/phonological Consonant/Vowel [CV] skeleton of words [1], [2], [34], [35], [36]), none of the current computational models of visual-word recognition and reading take the consonant-vowel status of letters into account in their ‘front-end’ and, instead, they assume that each letter in a word (whether consonant or vowel) is initially encoded in the same way (e.g., LTRS model [37]; overlap model [38]; spatial coding model [39]; Bayesian reader model [40]; SERIOL model [41]; overlap open-bigram model [42]; EZ-Reader model [43]; SWIFT model [44]). One potential limitation of all these models is their ‘static’ nature (i.e., they assume well-formed and unalterable set of parameters of visual-word recognition in a mature reading system), as they are not explicitly designed to deal with the dynamics of a developing reading system. Leaving aside the difficulty of creating a (formal) developmental model of visual-word recognition, it is critical to know what the important benchmarks are (i.e., what is there to simulate) and how they evolve in time (i.e., does the consonant bias appear at all levels of reading skill or only with skilled readers?). Clearly, developmental data on the consonant bias can contribute to a deeper understanding of the dynamics of language representation and processing while these abilities are still developing, thus helping to disentangle the mechanisms from which consonants and vowels are processed.

In the current series of experiments, we aimed to analyze the emergence of the consonant bias in reading. In particular, we examined the relative contribution of consonants and vowels at early stages of word recognition by conducting a series of experiments – using the procedure employed by New et al. [8] – with beginning (2^nd^ Graders) and intermediate (4^th^ Graders) European Portuguese (EP) developing readers. As a further control, we also conducted the parallel experiment with EP skilled adult readers (university students) - see [45]–[50] for a similar strategy. As in the New et al.'s experiment [8], we employed a masked priming paradigm and a highly common visual-word identification task (i.e., lexical decision). Di- and tri-syllabic target words (e.g., VASO [VASE] and PIRATA [PIRATE], respectively) selected from the children's lexical Portuguese database ESCOLEX (51) were preceded by a forwardly masked nonword prime (for 50 ms) which could be a consonant-preserving prime (e.g., vesu-VASO or pureto-PIRATA) or a vowel-preserving prime (e.g., zalo-VASO or gicala-PIRATA). We also included an identity priming condition (e.g., vaso-VASO or pirata-PIRATA) and an unrelated priming condition (e.g., tuce-VASO or bocelo-PIRATA) as further controls. As in the New et al. experiment, consonant and vowel position in words was controlled by using two distinct orthographic and phonological structures as defined by consonant (Cs) and vowel (Vs) order within the word in each syllable length. Thus, half of the target words started with a consonant (CVCV - e.g., VASO [VASE]; and CVCVCV words - e.g., PIRATA [PIRATE]) - hereafter CV words, and the other half started with a vowel (VCVC – e.g., AZUL [BLUE]; and VCVCVC words - e.g., ANIMAL [ANIMAL]) - hereafter VC words. We should note here that the consonant bias in the New et al. study (8) was observed both for CV (e.g., DIVA) and VC target words (e.g., OPUS). The only relevant difference with respect to the New et al. experiment was that here we employed the go/no-go variant of the lexical decision task (i.e., respond to words and refrain from responding to nonwords) rather than the yes/no variant. The go/no-go procedure was preferred because it provides faster and more accurate responses in young readers than the yes/no procedure, while tapping the same underlying processes [52], [53] - see also [54], for a mathematical model of the go/no-go task.

To sum up, Experiment 1 was conducted with EP adult skilled readers since previous experiments on consonant/vowel asymmetries were conducted in other languages (e.g., English, French, Spanish, etc.). To anticipate the findings, we found faster recognition times for target words preceded by consonantal information than for target words preceded by vocalic information (i.e., a consonant bias) regardless of the initial letter in the word (i.e., the magnitude of the consonant bias was similar for CV and VC words), hence replicating the New et al. [8] findings in EP. Thus, the critical question in this paper was whether the consonant bias would also be observed in beginning and intermediate developing readers. Experiment 2 was conducted with 2^nd^ Grade children and Experiment 3 with 4^th^ Grade children. If there is a developmental continuity between what is observed early in speech processing (at least in the acquisition of new words or in the recognition of familiar ones) and early in reading acquisition, one would expect an advantage of consonantal over vocalic information even at initial stages of reading acquisition.

## General Method

### Ethics Statement

The experiment was conducted with the approval of the Ethics Committee for Human Research of the Research Center on Psychology (CIPsi) at the University of Minho (Braga, Portugal). Written consent was obtained from the all participants in Experiment 1 (university students) or from all the parents of the developing readers in Experiment 2 (2^nd^ graders) and Experiment 3 (4^th^ graders).

### Materials

Sixty-four di- and tri-syllabic EP target words of four- and six-letters in length respectively were selected from the ESCOLEX database [51]. ESCOLEX is an EP grade-level lexical database that provides several word frequency statistics for 6- to 11-year-old children (1^st^ to 6^th^ Grade) computed from elementary textbooks - for details see [51]. Similarly to the New et al. experiment [8], half of the target words were CV words (16 CVCV and 16 CVCVCV words), whereas the other half were VC words (16 VCVC and 16 VCVCVC words). These two word structures were matched on word frequency (CV words, M = 150.04 occurrences per million words, range: 7.68–1,723.21; VC words, M = 147.52, range: 3.84–1,024.71), contextual diversity (i.e., proportion of textbooks in which the word appears; CV words, M = 0.29, range: 0.08–1.0; VC words, M = 0.33, range: 0.04–1.0), and number of orthographic (substitution-letter) neighbors (CV words, M = 1.28, range: 0–4; VC words, M = 1.16, range: 0–3) from the 1^st^ to the 4^th^ Grade (G_1_–G_4_) ESCOLEX level (all ts<1, ps>.52) – factors that have been shown to affect visual-word recognition in children [46], [47], [48], [55], [56]. Target words were presented in uppercase and were preceded by a nonword prime in lowercase that had the same orthographic and phonological structure as the target and could: (i) be the same as the target (i.e., identity priming condition - e.g., pirata-PIRATA); (ii) have preserved the same consonants as the target but with different vowels (i.e., consonant priming condition - e.g., pureto–PIRATA); (iii) have preserved the same vowels as the target but with different consonants (i.e., vowel priming condition - e.g., gicala-PIRATA); and (iv) not share any consonant and vowel with the target (i.e., unrelated priming condition - e.g., bocelo-PIRATA). We also created a set of 64 orthographically legal nonwords of four and six-letters with the same orthographic and phonological structure as the target words (e.g., NEPO, BOZUTA, AJOL, ILUNAL) for the purposes of the lexical decision task. These pseudowords had been created by replacing one or two letters in the medial and final positions of Portuguese words selected from the G_1_-G_4_ level of ESCOLEX with the same lexical characteristics as the experimental words. The manipulation for the nonword targets was the same as that for the word targets. Four lists of materials were created in order to counterbalance items in the four priming conditions (i.e., each target appeared once in each list, but each time in a different priming condition). Participants were randomly assigned to each list (six participants per list in each experiment). The list of materials (primes, target words and pseudowords for each experimental condition) used in the experiments is presented in File S1.

### Procedure

The experiment was run individually in a sound-proof booth for skilled adult readers (Experiment 1) and was conducted in groups of four children in a quiet room for developing readers (Experiments 2 and 3). Presentation of the stimuli and recording of responses were controlled by DMDX software [57]. Each trial consisted of a sequence of three visual events in black (18-pt Courier New font) on a white screen (15” monitor with a 60 Hz refresh). The first was a forward mask consisting of a row of hash marks (#) presented at the center of the screen for 500 ms. The mask was immediately replaced by the prime, in lowercase, for 50 ms (i.e., three refresh cycles). Thirdly, the target, in uppercase, replaced the prime and remained on the screen until participants' response or until 2500 ms had elapsed. Each of the three stimuli were centered in the screen (i.e., they all occupied the same position). Participants were asked to press the “sim”[yes] key if the letter sequence in uppercase was a Portuguese word, and to refrain from answering if the letter sequence was not a Portuguese word. Both speed and accuracy were stressed in the instructions. Participants were not informed of the presence of prime stimuli. Trial order was randomized for each participant. Prior to the 128 experimental trials, each participant received 18 practice trials with the same manipulation as that in the experimental trials. None of the participants reported having perceived the primes when asked after the experiment. The whole session lasted approximately 7–10 minutes with adults (Experiments 1 and 3) and 10–12 minutes with children (Experiments 2 and 3).

## Experiment 1 (adult skilled readers)

### Method

#### Participants

Twenty-four undergraduate students (M_age_: 20.6 years; range: 19–25; 21 female) from the University of Minho (Braga, Portugal) took part in the experiment in exchange for course credit. All participants had normal (or corrected-to-normal) vision and were native speakers of EP.

#### Materials and Procedure

See the General Method section.

### Results and Discussion

Incorrect responses (0.8% of the data for word targets) and response times (RTs) that exceeded two standard deviations from the participant's mean (5.0%) were excluded from the RT analysis. Repeated-measures of variance (ANOVAs) considering participants (F_1_) and item (F_2_) response latencies were conducted based on a 4 (prime type: identity, consonant-preserving, vowel-preserving, unrelated) × 2 (word structure type: CV vs. VC) × 4 (list: List 1, List 2, List 3, List 4) mixed design. In the F_1_ analyses, prime type and word structure were considered as within-subject factors and list as a between-group factor, while in the F_2_ analyses prime type was considered a within-subject factor, and word structure type and list as between-group factors. List was included in the analyses to remove the error of variance due to the four counterbalancing lists [58]. The percentage of errors was negligible (less than 1%) and it was not further analyzed. The mean correct RTs and error percentages from the subject analysis in each condition are presented in Table 1.

**Table 1:** Mean lexical decision times (RTs; in Milliseconds) and Percentage of Errors (% E) for consonant initial words (CV words), vowel initial words (VC words) and for all for the targets (all) in adults and developing readers (2^nd^ and 4^th^ Grade children).

Priming conditions	Adults						2^th^ Grade children						4^th^ Grade children					
	CV words		VC words		All		CV words		VC words		All		CV words		VC words		All	
	RT	% E	RT	% E	RT	% E	RT	% E	RT	% E	RT	% E	RT	% E	RT	% E	RT	% E
Identity	548 (11.9)	0.0 (0.0)	541 (13.0)	1.2 (0.8)	544 (11.4)	0.6 (0.4)	985 (35.6)	7.2 (1.9)	1,005 (32.8)	12.6 (2.4)	995 (31.8)	9.9 (1.6)	792 (35.3)	1.0 (0.7)	804 (33.6)	12.1 (2.3)	798 (32.9)	6.6 (1.1)
Consonant-preserving	592 (13.9)	0.5 (0.5)	579 (10.2)	2.4 (1.0)	586 (11.4)	1.4 (0.5)	1,061 (37.7)	5.4 (1.5)	1,025 (30.2)	13.3 (2.3)	1,043 (29.6)	9.3 (1.4)	814 (28.0)	2.3 (1.4)	852 (33.0)	9.5 (2.0)	833 (29.3)	5.9 (1.3)
Vowel-preserving	610 (14.1)	0.0 (0.0)	600 (14.0)	0.5 (0.5)	605 (12.7)	0.3 (0.2)	1,070 (36.9)	5.6 (1.6)	1,052 (31.1)	12.2 (2.1)	1,061 (28.7)	8.9 (1.4)	901 (35.0)	2.0 (1.1)	847 (40.8)	10.1 (1.7)	874 (34.1)	6.0 (1.1)
Unrelated	605 (10.8)	0.0 (0.0)	596 (13.9)	2.1 (1.1)	601 (11.9)	1.1 (0.5)	1,056 (33.2)	5.2 (1.8)	1,041 (27.6)	11.0 (2.2)	1,048 (28.8)	8.1 (1.5)	847 (27.5)	3.7 (1.4)	844 (37.5)	8.6 (1.5)	846 (31.3)	6.1 (1.2)

Standard errors are presented between brackets.

Note: Error rates for the pseudoword targets were 5.3, 9.0, and 6.2%, for the adult readers, 2nd graders, and 4th graders, respectively.

The ANOVA on the latency data revealed a main effect of prime type, F_1_(3,60) = 35.03, MSE = 1059, η^2^ = .64, p<.001; F_2_(3,168) = 28.22, MSE = 1782, η^2^ = .34, p<.001. Leaving aside the unsurprising advantage of the identity condition over the other priming conditions, planned comparisons revealed a 19 ms advantage of the consonant-preserving condition over the vowel-preserving condition, F_1_(1,20) = 5.75, MSE = 1603, η^2^ = .22, p = .026; F_2_(1,56) = 8.02, MSE = 1740, η^2^ = .13, p = .006 – note that the vowel-preserving condition behaved similarly to the unrelated condition (605 vs. 601 ms, respectively). The ANOVA on the RT data also revealed that CV words were recognized slightly slower than VC words (9.8 ms less), although this effect was far from significant in the analysis by items, F_1_(1,20) = 5.52, MSE = 808, η^2^ = .22, p = .029, F_2_<1- this is probably the result of a speed/accuracy trade-off (i.e., error rates for VC words were 1.7% whereas the error rates for CV words were 0.1%). There were no signs of an interaction between the two factors, both Fs<1, ps>.51.

As expected, a consonant bias (i.e., faster responses to pureto-PIRATA than to gicala-PIRATA) occurs during the early stages of visual-word recognition with Portuguese adult readers, replicating the results reported by New et al. [8] with French adult readers (i.e., in a syllable-timed language). Likewise, as in the New et al. experiment the magnitude of the consonant bias (i.e., Vowel-preserving primes minus Consonant-preserving primes) was similar for CV words (e.g., PIRATA: 18 ms) and for VC words (e.g., ANIMAL: 21 ms) (see Table 1). Although – as a reviewer pointed out – there seemed to be a small speed-accuracy trade-off for the CV words (−0.5%) and for VC words (−1.9%), this was due to a very small set of words (60 out of the 64 words yielded no errors in the experiment). In fact, if we exclude these four words from the latency analyses, the pattern of data was essentially the same as that presented here (i.e., an overall 19 ms advantage of the consonant-preserving over the vowel-preserving condition). Thus, the present experiment provides additional support to the idea that consonants have a privileged role in lexically-related processes, a result that has been systematically observed in other languages with skilled-adult readers [3]–[12] – note that Portuguese is a language with a greater consonant/vowel repertoire asymmetry than Spanish, but smaller than English and French.

Once the presence of a consonant bias in the early stages of visual-word recognition in Portuguese has been established, the next question is whether beginning (2^nd^ graders) and intermediate (4^th^ graders) Portuguese developing readers would also show the same advantage at early stages of visual-word recognition. If consonants facilitate visual-word recognition to a greater extent than vowels even in beginning readers (2^nd^ graders), this would signal a developmental continuity between what is observed in speech processing in infants and preschool children and the initial stages of reading acquisition. Furthermore, it would also strengthen the idea of a fundamental basic distinction between consonants and vowels in language processing, as Nespor et al. [29] proposed.

## Experiment 2 (2^nd^ graders)

### Method

#### Participants

Twenty-four 2^nd^ Grade children (M_age_: 7.5 years; range: 7–8; 12 female) participated voluntarily in the experiment. The children came from above-average socioeconomic backgrounds and attended a private school in Porto, Portugal. All participants had normal (or corrected-to-normal) vision and were native speakers of EP. None of them had any sensory, neurological, or learning disabilities. All participants had normal scores on standardized reading tests, adjusted to their grade level. The experiment took place at the end of the school year.

#### Materials and Procedure

See the General Method section.

### Results and Discussion

Incorrect responses (9.0% of the data for word targets) and latencies that exceeded two standard deviations from the participant's mean (4.4%) were excluded from the RT analysis. The statistical analyses were parallel to those in Experiment 1, except that the error data were now also analyzed. The mean correct RTs and error percentages from the subject analysis in each condition are presented in Table 1. The ANOVA on the latency data revealed a main effect of prime type, F_1_(3,60) = 3.87, MSE = 10438, η^2^ = .13, p = .016; F_2_(3,159) = 6.08, MSE = 13205, η^2^ = .10, p = .001, reflecting an advantage of the identity condition over all other priming conditions, all ps<.001 – note that there were no significant differences among consonant, vowel, and unrelated conditions (all Fs<1). Neither the effect of the word structure (CV vs. VC) nor the interaction between the two factors approached significance, both Fs<1, both ps>.48. The ANOVA on the error rates revealed a main effect of word structure in the analysis by subjects, F_1_(1,20) = 17.29, MSE = 114.9, η^2^ = .46, p<.001; F_2_(1,56) = 2.66, MSE = 986, η^2^ = .05, p = .107. This effect showed that 2^nd^ graders made more errors on VC than on CV words (12.3% vs. 5.9%, respectively). Neither the effect of prime type nor the interaction between the two factors approached significance, both Fs<1, both ps>.70.

Results from the present experiment with Portuguese beginning readers (2^nd^ graders) only revealed a masked identity priming effect - see also [46] for evidence of masked identity priming effects in the lexical decision task with 2^nd^ graders. Even though the target words were highly familiar in children's lexicons (M = 178.8 occurrences per million words in the G_1_–G_4_ grade-level in the ESCOLEX database), neither consonant- nor vowel-preserving nonword primes produced a significant advantage over the unrelated priming condition (see Table 1). The lack of priming effects with preserving-consonant (or preserving-vowel) prime conditions is probably due to the fact that the orthographic processing system in 2^nd^ graders is not yet sufficiently tuned in order to optimize the information provided by the prime to help word recognition – see [26]–[28] for developmental models of reading acquisition.

Under these circumstances, it is likely that the brief presentation of consonant- or vowel-preserving primes (50 ms) that only shared half of the letters with the target word (e.g., pureto-PIRATA or gicala-PIRATA) was not sufficient to produce any significant priming effects beyond an overall identity priming effect. Encoding processes are slower in developing readers than in adult skilled readers - see [59] for evidence with the diffusion model. It is also important to note here that the previous demonstrations of masked priming effects with beginning readers involved primes that only differed from the target word by one substituted letter (e.g., rlay-PLAY) or by the transposition of one adjacent letter (e.g., lpay-PLAY) [45], [46], [47], and thus contained much more target information than the primes in our study. The only exception comes from Goikoetxea's study [60] who reported masked syllable priming effects in Spanish for CVCV words (e.g., pato-PANA [duck-CORDUROY] vs. foto-PANA [photo-CORDUROY]) in 2^nd^ graders. However, in this case prime duration (120 ms) was more than twice as long as the one that we employed here (50 ms) (i.e., the primes were visible).

Thus, the absence of any other significant priming effects (besides identity priming effects) in the present experiment must be taken with caution since the present manipulation was probably too subtle to have an effect in beginning readers. Nonetheless, as exposure to print increases, and the orthographic processing system develops, it is reasonable to think that children are progressively able to extract the information presented in the prime in a more effective way, which allows for subtler masked priming effects to be observed. Therefore in Experiment 3, we explored whether the consonant bias would be noticeable later in reading acquisition with 4^th^ Grade children.

## Experiment 3 (4^th^ graders)

### Method

#### Participants

Twenty-four 4^th^ Grade children (M_age_: 9.9 years; range: 9.5–10.5; 13 female) from the same private school as the children from Experiment 2 participated in this experiment. All participants had normal (or corrected-to-normal) vision and were native speakers of EP. None of them had any sensory, neurological, or learning disabilities. All participants had normal scores on standardized reading tests, adjusted to their grade level. As in Experiment 2, this experiment took place at the end of the school year.

#### Materials and Procedure

See the General Method section.

### Results and Discussion

Incorrect responses (6.2% of the data for word targets) and response times that exceeded two standard deviations from the participant's mean (4.75%) were excluded from the RT analyses. The statistical analyses were parallel to those in Experiments 1 and 2. The mean correct RTs and error percentages from the subject analyses are presented in Table 1.

The ANOVA on the latency data revealed a main effect of prime type, F_1_(3,60) = 7.18, MSE = 6661, η^2^ = .26, p<.001; F_2_(3,165) = 8.26, MSE = 8110, η^2^ = .13, p<.001. As in Experiment 1, and leaving aside the unsurprising advantage of the identity condition over the other priming conditions, 4^th^ Grade children showed an overall 41 ms advantage of the consonant-preserving condition over the vowel-preserving condition (see Table 1). The main effect of word structure was not significant, both Fs<1, ps>.59. Importantly, CV and VC words produced a different pattern of priming effects, as reflected by the interaction between the two factors, F_1_(3,60) = 2.92, MSE = 6207, η^2^ = .13, p = .041; F_2_(3,165) = 4.23, MSE = 8110, η^2^ = .07, p = .007. This interaction revealed that in VC words there were no signs of a difference between the consonant-preserving and the vowel-preserving priming conditions, both Fs<1 – note that the consonant-preserving (852 ms) and the vowel-preserving (847 ms) priming conditions behaved similarly to the unrelated condition (844 ms) (see Table 1). That is, only identity primes showed a significant advantage over the other conditions – similarly to the pattern of data observed with 2^nd^ graders (Experiment 2). However, in CV words there was a statistically significant advantage of 87 ms of the consonant-preserving prime condition over the vowel-preserving prime condition, F_1_(1,20) = 19.43, MSE = 4753, η^2^ = 49, p<.001, F_2_(1,28) = 24.59, MSE = 5507, η^2^ = .47, p<.001. Importantly, the difference between the consonant-preserving and vowel-preserving conditions was not just due to the presence of faster response times in the consonant-preserving condition relative to the unrelated condition (33 ms), but also to the longer response times in the vowel-preserving condition relative to the unrelated condition (−54 ms), F_1_(1,20) = 6.15, MSE = 6977, η^2^ = 24, p = .02; F_2_(1,28) = 8.01, MSE = 5792, η^2^ = .22, p = .009.

The ANOVA on the error data revealed a main effect of word structure F_1_(1,20) = 70.97, MSE = 41.4, η^2^ = .78, p<.001; F_2_(1,56) = 4.53, MSE = 858, η^2^ = .07, p = .038. As in Experiment 2 with 2^nd^ graders, this effect showed that 4^th^ Grade children made more errors for VC than for CV words (10.1% vs. 2.3%, respectively). Neither the effect of prime type nor the interaction between the two factors approached significance, both Fs<1.96, both ps>.12.

The present experiment with 4^th^ Grade children revealed an advantage of consonants over vowels at early stages of visual-word recognition, hence establishing the emergence of a consonant bias at this stage of reading acquisition with young readers. Importantly, the consonant bias in 4^th^ graders was restricted to CV words. Another finding that deserves some consideration has to do with the fact that the consonantal advantage observed in CV words was not only due to a 33 ms facilitative effect of the consonant-preserving condition relative to the unrelated condition, but also to an inhibitory effect of the vowel-preserving condition relative to the unrelated condition. Fourth graders were 54 ms slower in recognizing CV words preceded by a vowel-preserving prime than by an unrelated prime. Although somehow unexpected, this vocalic inhibitory effect is not entirely novel. Indeed in News et al. 's study [8] with French adult skilled readers, the authors reported a nonsignificant 10 ms disadvantage of the vowel-preserving priming condition over the unrelated condition for CV words in the latency data (and a disadvantage of 3.9% in the error data). This tendency reached statistical significance in a recent study by New and Nazzi [9] that examined the orthographic or phonological/lexical nature of the consonantal bias. The authors found a significant disadvantage of the vowel-related priming condition relative to the unrelated condition for CV and VC words when the prime was presented for 66 ms, and also when the prime was presented for 50 ms and followed by a 16 ms postmask (i.e., the stimulus-onset asynchrony [SOA] was 66 ms). New and Nazzi [9] argued that this inhibitory effect from primes sharing vowels with the target was likely to be due to some lexical activation from the number of consonant or vowel skeleton neighbors shared between the target and its consonant and vowel primes respectively (see also [61] for evidence of shared neighborhood effects with one-letter different primes). To further examine this issue we computed as [9], the number of vocalic skeleton neighbors (e.g., VASO [vase]: *A*O, taco, nado, dado, ralo, lavo, raro, favo, falo, raso etc.; AZUL[blue]: A*U*, aqui, atum, agua, amuo, amua, alua) and the number of consonantal skeleton neighbors (e.g., VASO[vase]: V*S*, visa; AZUL[blue]: *Z*L, none) for each of the target words in the ESCOLEX database [51]. For CV words, the mean number of vowel and consonant skeleton neighbors was 30.0 and 1.6, respectively, whereas for VC words, the mean number of vowel and consonant skeleton neighbors was substantially smaller: 6.6 and 1.5, respectively. Assuming that early in development the child's orthographic processing system is coarsely tuned and has not had enough time to “learn” how to optimize the mapping of letter representation onto semantics using the best quality information in the stimulus, it is possible that it tolerates to a greater extent the “noise” introduced by incongruent primes - as observed in the decrease of the magnitude of the substituted-letter and transposed-letter priming effects as a function of children's age and reading skills [28], [47]. Therefore, in the present study, vowel-preserving primes like zalo-VASO or gicala-PIRATA would activate a large number of lexical candidates – and clearly more than consonant-preserving primes like vesu-VASO or pureto-PIRATA, leading to the vowel inhibitory priming effect observed. Under the assumption of inhibitory connections at the lexical level, the large number of skeleton neighbors activated by the vowel-preserving primes in CV words may slow down target recognition. Evidence of lexical inhibition from shared neighbors with developing readers was also reported by Goikoetxea [60] using syllabic word neighbors as primes. Specifically, at a 120 ms prime exposure, the author found longer response times for pato-PANA [duck-CORDUROY] than for the control foto-PANA [photo-CORDUROY]. However as age/exposure to print increases and the orthographic system becomes more fine-tuned - in order to allow for an efficient word recognition process within a larger and more competitive lexicon (i.e., a lexicon with more and more similar words) [28], [47], [59] - it is possible that the lexical competition from the skeleton neighbors diminishes thereby leading to a decrease of the inhibitory effects produced by loosely related word units. Future research should be conducted in order to test directly the effect of shared skeleton neighborhood in developing readers.

Finally, for VC words neither the consonant- nor the vowel-preserving primes produced any significant priming effects (besides the identity priming effect) in 4^th^ graders, mimicking the pattern of data observed with 2^nd^ graders with CV and VC words. Taken together, these findings suggest that the consonant bias emerges at intermediate levels of reading acquisition (4^th^ graders) and gradually, that is it is firstly observed in CV words (and not in VC words).

## General Discussion

Recent research with skilled adult readers has consistently revealed an advantage of consonants over vowels (i.e., a “consonant bias”) at early stages of visual-word recognition and reading [1]–[12]. The present masked-priming lexical decision experiments extend this line of research to developing readers (2^nd^ and 4^th^ graders) by examining the emergence of consonant/vowel asymmetry at early stages of visual-word recognition. The main findings can be summed up as follows: (i) an advantage of consonant-preserving primes over vowel-preserving primes was observed with college-aged readers in Portuguese, thus extending previous experiments with adult skilled readers in other alphabetic languages (e.g., English, French, Spanish) to Portuguese; (ii) an advantage of consonant-preserving primes over vowel-preserving primes occurred for 4^th^ graders but not for 2^nd^ graders (i.e., the consonant bias emerges at a later, intermediate stage of reading acquisition); and (iii) the advantage of consonant-preserving primes over vowel-preserving primes was observed for both CV and VC words in adult skilled readers, though restricted to CV words in 4^th^ graders (i.e., the consonant bias emerges gradually in reading acquisition being modulated by the CV word structure).

Contrary to what was observed in the vast number of studies conducted with young children and preschoolers in speech processing [13]–[25] and with adult skilled readers in visual-word recognition [1]–[12], the present masked priming experiments reveal that in early stages of reading acquisition (2^nd^ Grade children) neither consonant-preserving primes (e.g., pureto-PIRATA) nor vowel-preserving primes (e.g., gicala-PIRATA) had a significant effect upon the processing of the target words relative to an unrelated condition. At this stage, only identical primes facilitated the recognition of the target words. The absence of any significant effect of consonant- and vowel-preserving primes in beginning readers may well stem from the fact the child's orthographic processing system is coarsely tuned and has not had enough time to “learn” how to optimize the mapping of letter representation onto semantics using the best quality information in the stimulus [28], [47], [59]. The technique used in this experiment (i.e., masked priming) taps into rapid and automatic visual-word recognition processes, and the standard 50 ms duration of prime exposure was probably insufficient to cause a reliable effect on word recognition with partial primes. We must keep in mind that only half of the letters in the target word were preserved in the consonant-preserving primes (e.g., pureto-PIRATA) or in the vowel-preserving primes (e.g., gicala-PIRATA) and there is modeling evidence that shows that the quality of extraction information from word stimuli (i.e., “drift rate” in a diffusion model) is lower in young readers than in adults [62]. Prior demonstrations of masked form priming effects with beginning readers employed either a greater perceptual overlap between primes and targets - such as one-letter different primes and/or transposed-letter primes [28], [45], [47] or a longer time exposure of the prime (120 ms) [60]. Further research is necessary to examine in detail the interplay between prime duration and age/reading skills across different manipulations (e.g., identity primes, partial primes, and unrelated primes) with the masked priming paradigm.

Nevertheless, as age/reading skills increase, young readers process printed words more accurately and rapidly and the orthographic system is able to maximize the information provided by the stimulus in order recognize words. This would readily explain the emergence of the relative advantage of consonant-preserving primes over vowel-preserving primes in 4^th^ graders (around 9-10 year olds). Importantly, the advantage of consonants over vowels in visual word recognition at this stage of reading acquisition (intermediate) was observed for CV words (i.e., words like VASO [VASE] and PIRATA [PIRATE]), but not for VC words (i.e., words like AZUL [BLUE] and ANIMAL [ANIMAL]). Note that in this study, CV and VC target words were matched in lexical frequency, contextual diversity, and number of orthographic neighbors (see the Materials section). A possible explanation for this unexpected effect may rely on the overall familiarity with the CV skeletal structure of words. In Romance languages such as Portuguese, words with a CV structure are more frequent than words with a VC structure [63]. An a posteriori analysis of the distribution of the orthographic word structures in the G_1_–G_4_ level of the ESCOLEX children's database [51] supports this idea as it revealed that words with a CV structure occur in 82.5% of all four- and six-letter wordforms, whereas words with a VC structure occur only in 11.6% of all four- and six-letter wordforms. Moreover, words with a CV structure occur not only in more words in young readers' lexicons but also in words with a higher frequency of occurrence (the token frequency of CV words is 69,945.97 per million words, while the token frequency for VC is 8,280.66). Therefore, the lack of a priming effect observed in 4^th^ graders for VC words could be due to the fact that the sublexical activation generated from the prime was reduced when the word structure was less familiar. Importantly, as reading skills and exposure to printed words increase, this sensitivity to the word's orthographic structure at early stages of visual-word processing tends to vanish and the consonant bias extends to other less familiar word structures. Indeed, both VC and CV word structures produced a similar consonant bias in adult skilled-readers (see Experiment 1; see also [8]) which might signal that the orthographic system reached its maturity and is able to maximize the information provided in the stimulus that best helps to identify words.

Before considering the implications of the consonant bias for models of visual-word recognition in terms of an abstract CV skeleton in which consonants and vowels play different (functional) roles, it may be important to examine an alternative (more perceptual) explanation. It might be argued that consonants and vowels behave differently during lexical access because of the different characteristics in letter shape. While many consonant letters have an ascending (e.g., b, t, d, k, l) or descending (e.g., j, q, g, p) shape, all vowels are neutral letters (e.g., a, u, e). Therefore, the detection of an ascending/descending letter offers unambiguous cues of the consonant/status of the letter and it may help word processing. Although Grainger and Dufau [64] used this reasoning to explain why lowercase words are identified more rapidly than uppercase words [65], [66], it can be easily generalized to the processing of the lowercase stimuli in masked priming (i.e., the ascending/descending shape of the lowercase primes could have provided a head start). To minimize the role of the letter-shape information in the consonant bias phenomenon, we conducted a replication of Experiment 1 (40 Portuguese university students; M_age_: 20.2 years; range: 18–29; 32 female) in which the primes were presented in uppercase and targets were presented in lowercase [67] for a comparison between lowercase/uppercase vs. uppercase/lowercase prime-target pairs). Keep in mind that uppercase stimuli do not have a specific letter shape, so that the letter-shape of the prime stimuli does not provide any clues on the consonant/vowel status of the letters [49]. Results revealed exactly the same pattern as in Experiment 1. In particular, we found a sizeable consonant bias effect (17 ms in the latency data [0.3% in the error data]; the parallel response time advantage in Experiment 1 was 19 ms). Therefore, the consonant bias that has been found in Experiment 1 (or in previous research) is not due to some uncontrolled effect of the ascending/neutral/descending nature of the letters in the lowercase primes. This finding is also consistent with previous evidence that has failed to find consistent effects of letter/word shape with normal readers [49], [68].

What are the implications of these findings for (developmental) computational models of visual word recognition? The presence of a robust consonant bias not only for adult readers, but also for young readers does suggest that some form of CV skeleton needs to be added to current computational models of visual-word recognition that do not encode the letters as consonants or vowels (e.g., LTRS model [37]; overlap model [38]; spatial coding model [39]; Bayesian reader model [40]; SERIOL model [41]; overlap open-bigram model [42]; EZ-Reader model [43]; SWIFT model [44]). Furthermore, we have shown that the consonant bias in developing readers is modulated by the familiarity of the words' structure (i.e., it emerges primarily in the canonical CV structure and is afterwards generalized to VC structures in adult skilled readers), thus suggesting that the study of the consonant bias in visual-word recognition should not be dissociated from the linguistic experience with printed words and with the statistical regularities of the language.

One remaining issue at the theoretical level here is at which level of processing the CV skeleton takes place, whether at a very early orthographic level or when phonology starts playing a role. Of particular interest here would be to explore the consonant bias in word structures in which there is a mismatch between the orthographic and the phonological mapping, as in cases of consonantal clusters (e.g., words like “strong”[strσŋ] a CCCVCC orthographic structure and a CCCVC phonological structure) and/or in vowel hiatus words from a developmental perspective (e.g., words like “chaos”[′keΙσs] a CCVVC orthographic structure and a CVVC phonological structure – as recently used with adult readers [36]). Clearly, further developmental research should explore to what extent the consonant bias is linked to the phonological (or lexical) characteristics of the word-processing system during visual-word recognition and reading by using techniques that tap the time course of processing such as ERP recording and/or eye movement paradigms.

To conclude, the present findings set some constraints on future (developmental) models of visual-word recognition that incorporate a Consonant/Vowel skeleton [1], [34], [35]. Firstly, consonantal information produces greater facilitation than vowel information (i.e., a “consonant bias”) at early stages of visual-word recognition with young readers, at least from 4th Grade onwards. Secondly, the emergence of the consonant bias is modulated by the familiarity of the CV skeleton structure – it occurs firstly with the most familiar CVCV/CVCVCV word structure. Further research is necessary to examine in greater detail the time course of consonantal vs. vocalic processing of young readers during sentence reading.

## Supporting Information

File S1 **Appendix A: List of stimuli in the experiment.**
(DOCX)
