# Supplementary material for: Tracking the Emergence of the Consonant Bias in Visual-Word Recognition: Evidence with Developing Readers
Source: PLoS One. 2014 Feb 11;9(2):e88580. doi: 10.1371/journal.pone.0088580 (PMC3921185; doi:10.1371/journal.pone.0088580)
Supplement: File S1 — Appendix A: List of stimuli in the experiment. (DOCX) [file pone.0088580.s001.docx]

Appendix A. List of stimuli in the experiment

The stimuli are presented in quintuplets in the following order: TARGET, identity prime, consonant-preserving prime, vowel-preserving prime, and unrelated prime

CVCV/CVCVCV words: PERU, peru, pora, gemu, bosa; VASO, vaso, vesu, zalo, tuce; NETO, neto, nita, rexo, sufa; NOME, nome, namu, pore, ciba; ZONA, zona, zeno, xora, fuge; DOCE, doce, duci, bose, tisa; JOGA, joga, jigo, pofa, tile; RISO, riso, resa, nimo, dume; MIMO, mimo, mamu, biro, zula; LUME, lume, loma, vube, geto; JIPE, jipe, jopa, fise, hota; BASE, base, buso, tane, dova; POVO, povo, puva, zoco, rila; BODE, bode, bida, moge, duta; NABO, nabo, niba, pafo, rise; LOJA, loja, lije, zoba, mile; PIRATA, pirata, pureto, gicala, bocelo; RECADO, recado, rucoda, sezabo, tupela; CEBOLA, cebola, cibelo, fesota, vocuro; MENINO, menino, manuna, deliso, hocase; COLETE, colete, calita, hoseme, jafora; JANELA, janela, jonilo, tazema, besuro; PANELA, panela, pinulo, safema, diroco; PILOTO, piloto, pulite, bisoco, sacema; CARECA, careca, corico, valema, tosufo; GAVETA, gaveta, govite, malexa, hipuse; CANECA, caneca, cunico, haseta, polimo; CIDADE, cidade, cedudo, gipale, boguma; GULOSO, guloso, golisa, jumoto, camite; TECIDO, tecido, toceda, leriso, gajula; CASOTA, casota, cosite, pazola, picemo; CABELO, cabelo, cobila, sameto, rucima; LEJA, leja, lujo, zesa, pigo; NIRE, nire, naro, hige, dafo; BOZO, bozo, bize, pogo, sate; NEPO, nepo, nipa, lefo, zute; VUPO, vupo, vape, luno, jiza; MUPE, mupe, mipa, cuve, sivo; NASO, naso, nuse, lapo, jine; JUTA, juta, jote, huca, mevo; VUBO, vubo, veba, fupo, bare; DUTO, duto, deta, zupo, jime; HIVE, hive, huvo, nite, gupo; ZAVO, zavo, zive, jano, dipe; JUJE, juje, joja, lune, vico; DIFO, difo, dufa, ribo, sesa; SAJE, saje, sijo, mafe, nido; RURA, rura, rore, guna, fice; BOZUTA, bozuta, bizeto, jolusa, ceriso; NIVESO, niveso, navusa, fitepo, daruta; PECOLE, pecole, piculo, lefope, situfa; LURIFO, lurifo, larefa, putigo, rageva; SELIBO, selibo, saluba, decipo, pucala; GABINA, gabina, gobune, tariga, lofute; RULIFE, rulife, ralofa, vujile, pasoga; TIRODA, tiroda, terude, zilota, geluco; MUTASA, mutasa, motese, culapa, rodice; FIZUTE, fizute, fozata, sirule, lavoga; BORIPA, boripa, burepe, nogila, nulete; SAROFO, sarofo, surifa, matogo, fitula; SORIJA, sorija, seruje, rosifa, zevure; VIJODO, vijodo, vujeda, tipoto, hufena; FUVELO, fuvelo, fivala, zutero, jibara; HURATA, hurata, hirote, lupana, bicole

VCVC/VCVCVC words: ANEL, anel, onil, arez, esor; UVAS, uvas, evis, unar, ipor; UNIR, unir, anor, umis, asel; ANOS, anos, inas, arol, emaz; AGIR, agir, uger, apim, ujel; AZUL, azul, izol, afur, esis; AZAR, azar, ozer, anam, omel; USAR, usar, esir, utaz, otim; ORAL, oral, erul, omas, icez; ATUM, atum, otam, afus, ocem; AMAR, amar, omer, anaz, etos; ASAS, asas, esos, araz, obol; ARES, ares, oris, amez, ilom; ELOS, elos, ilas, etom, icaz; ODOR, odor, ader, onoz, ebas; AVES, aves, uvos, aber, ogil; EFICAZ, eficaz, ofecuz, elimar, avoril; AJUDAR, ajudar, ojedir, aculam, oretis; ACABAR, acabar, ecobir, azasal, oletim; AMIGOS, amigos, umegas, arisor, italez; USADOS, usados, isudes, ucaboz, amival; IMAGEM, imagem, amogim, ifatel, obinor; EDITOR, editor, udater, elisoz, ocazas; ORIGEM, origem, aregim, obirez, ucasur; ALUNOS, alunos, elanis, asurol, otapim; AMADOR, amador, emider, asalom, uposel; EVITAR, evitar, uvator, elimaz, asobiz; ANIMAL, animal, unomel, azicas, orober; AVISOS, avisos, evusas, afilom, eranez; ADORAR, adorar, idurer, atocam, opisen; APEGAR, apegar, ipugor, aresal, ubacen; IDOSOS, idosos, adesus, ivocoz, afetir; IMEL, imel, omal, igez, ucar; OTOS, otos, itas, ofol, anez; APIR, apir, oper, abis, ejus; UCAS, ucas, ocis, ulaz, oger; ACIR, acir, icer, atis, olus; ULIM, ulim, alom, upil, ecas; ITOR, itor, otur, icos, abul; OCER, ocer, ucir, orel, ixas; IVOL, ivol, evul, iros, ezus; EXIL, exil, oxal, ezis, ugam; ERUR, erur, iror, epus, udas; AJOL, ajol, ujil, acoz, epes; UROS, uros, eris, utol, ifel; OSES, oses, isos, odez, ubil; APUL, apul, opil, acus, onas; UFOS, ufos, afes, utom, icaz; ILUNAL, ilunal, alenol, idutas, etevos; ENITAR, enitar, inutor, ebilas, ujolos; AVEJOR, avejor, ovijur, axelos, odibes; OCIFOS, ocifos, acefus, onipol, unejaz; ANUROS, anuros, inares, acugoz, edilem; URETAZ, uretaz, aritoz, utejas, oticol; OSUNAR, osunar, esiner, ojufas, utezos; OVIDEL, ovidel, avudal, ocites, azutar; UDILOS, udilos, edulas, ugitol, elafam; AJICOS, ajicos, ujacas, alitoz, uzetel; OXEFAR, oxefar, uxifur, oletas, iridus; IPUTOL, iputol, apetal, imugos, esacas; ALAFOS, alafos, ilofes, agaloz, ogitez; OFEPAR, ofepar, ufipur, ovelas, inules; ENACAR, enacar, inucor, etalas, ubipel; AMEPES, amepes, omipas, afetel, utiboz
